# The *Pseudoalteromonas* multipartite genome: distribution and expression of pangene categories, and a hypothesis for the origin and evolution of the chromid

**DOI:** 10.1093/g3journal/jkab256

**Published:** 2021-07-21

**Authors:** Cecilie Bækkedal Sonnenberg, Peik Haugen

**Affiliations:** Department of Chemistry and Center for Bioinformatics (SfB), Faculty of Science and Technology, UiT The Arctic University of Norway, Tromsø N-9037, Norway

**Keywords:** *Pseudoaltermonas*, pangenome, multipartite, chromid, Alteromonadales, secondary replicons

## Abstract

Bacterial genomes typically consist of one large chromosome, but can also include secondary replicons. These so-called multipartite genomes are scattered on the bacterial tree of life with the majority of cases belonging to Proteobacteria. Within the class gamma-proteobacteria, multipartite genomes are restricted to the two families *Vibrionaceae* and *Pseudoalteromonadaceae.* Whereas the genome of vibrios is well studied, information on the *Pseudoalteromonadaceae* genome is much scarcer. We have studied *Pseudoalteromonadaceae* with respect to the origin of the chromid, how pangene categories are distributed, how genes are expressed relative to their genomic location, and identified chromid hallmark genes. We calculated the *Pseudoalteromonadaceae* pangenome based on 25 complete genomes and found that core/softcore are significantly overrepresented in late replicating sectors of the chromid, regardless of how the chromid is replicated. On the chromosome, core/softcore and shell/cloud genes are only weakly overrepresented at the chromosomal replication origin and termination sequences, respectively. Gene expression is trending downwards with increasing distance from the chromosomal *oriC*, whereas the chromidal expression pattern is more complex. Moreover, we identified 78 chromid hallmark genes, and BLASTp searches suggest that the majority of them were acquired from the ancestral gene pool of Alteromonadales. Finally, our data strongly suggest that the chromid originates from a plasmid that was acquired in a relatively recent event. In summary, this study extends our knowledge on multipartite genomes, and helps us understand how and why secondary replicons are acquired, why they are maintained, and how they are shaped by evolution.

## Teaser

Although several hypotheses have been proposed, the significance of secondary genetic elements remains uncertain. In an effort to extend our current knowledge on multipartite genomes we studied how different gene categories are distributed and expressed in *Pseudoalteromonas*. Our data support that secondary replicons are open and flexible in nature, and likely serve a variety of specialized roles in bacteria. This work also adds to the growing body of evidence demonstrating that bacterial cells are highly organized at all levels.

## Introduction

Multipartite genomes are recognized by the concurrent presence of multiple replicons, *i.e.*, cells contain one or more large replicons in addition to the chromosome (Harrison *et al.* 2010). The majority of bacteria with multipartite genomes are associated with high tolerance to abiotic stresses, or are associated with animals, human, or plants as pathogens or symbionts (Misra *et al.* 2018). This observation, in addition to other data, has prompted scientists to hypothesize that multipartite genomes play crucial roles in the successful spread and establishment of bacteria into a broad range of ecological niches (Heidelberg *et al.* 2000). A striking and well-studied example is the bacterium *Vibrio fischeri*. Some strains colonize the light-emitting organ of squid (*e.g.*, the Hawaiian bobtail squid *Euprymna scolopes*), and produce bioluminescent light that enables the host to evade predators by counter-illumination (Soto and Nishiguchi 2014). Other strains are in contrast pathogens, which apparently is made possible due to the presence of a gene capture system, a superintegron, and pathogenicity islands located on the chromid (Soto and Nishiguchi 2014; diCenzo *et al.* 2019). Therefore, a link between the two-replicon genome architecture and the bacteria’s lifestyle has been suggested. The reality is, however, that although carrying one or more extra replicon may promote new opportunities for a bacterium to move into new niches, other bacteria thrive in the same environment without additional large replicons, thus demonstrating that multipartite genomes are probably not necessary to succeed in that environment. Our general understanding of the origin, evolution, and functional roles of multipartite genomes remains fragmented, and multiple other equally likely hypotheses have been proposed to explain their existence. For example, carrying genes on more than one large replicon allows for replicon-specific gene dosage, and consequently replicon-specific gene expression regulation (*e.g.*, Couturier and Rocha 2006; Dryselius *et al.* 2008). Also, it has been suggested that the presence of multiple replicons allows bacteria to contain larger genomes, and may reduce the time required to complete replication thus allowing for rapid cell growth and division (diCenzo *et al.* 2014). Finally, we recently proposed a hypothesis that the presence of two large replicons allows for intracellular spatial separation of different categories of genes, and that there is a link between the skewed gene placement and their function (Sonnenberg *et al.* 2020). The underlying reason for the separated distribution of gene categories in 3D is however likely very complex, and not easy to dissect.

Given that one or more of the hypotheses above are correct, then it is not surprising that bacteria with multipartite genomes are indeed widely distributed. They are as of today found scattered across the bacterial kingdom, into 6 of 81 phyla listed in the NCBI taxonomy system (*i.e.*, Proteobacteria, Bacteriodetes, Actinobacteria, Firmicutes, Deinococcus-Thermus, and Spirochaetes) (diCenzo and Finan 2017). It is highly likely however that more examples of multipartite genomes remain to be discovered, especially when considering that the number of complete genomes in the databases is still relatively low (∼23,000), and dominated by Proteobacteria (57%) and Terrabacteria (34%). One hundred one of 127 multipartite genomes group within the phylum Proteobacteria (diCenzo and Finan 2017), which means that the remaining 26 genomes are distributed among five other phyla. Inside the class gamma-proteobacteria, multipartite genomes are restricted to *Vibrionaceae* and *Pseudoalteromadaceae.* The multipartite genome of *Vibrionaceae* consists of one large circular chromosome (4.1−2.7 Mb) known as Chromosome 1 (Chr 1), and one smaller circular replicon (2.3−0.7 Mb) knows as Chromosome 2 (Chr 2), hereafter referred to as the *Vibrionaceae* chromid, in accordance with the nomenclature by Harrison *et al.* (2010). Replication of the *Vibrionaceae* chromosome and chromid is precisely coordinated by a mechanism that is partly understood. Briefly, when the replication fork approaches *crtS* (chromid replication triggering site) in Chr 1, a hitherto unknown mechanism triggers replication of the chromid (Val *et al.* 2016; Kemter *et al.* 2018), there is a brief pause in Chr 1 replication before the cycle ends in a synchronized termination of replication. In fast-growing bacteria, such as *Vibrio cholerae* and *Vibrio natriegens*, replication results in higher gene copy numbers of genes surrounding the origin of replication of Chr 1 (*ori1*) (this is known as “gene dosage effect”). Consequently, expression of genes typically decreases with increasing distance from *ori1* (Dryselius *et al.* 2008; Toffano-Nioche *et al.* 2012). This correlation does not necessarily apply to slow-growing bacteria, or fast-dividing bacteria grown under poor (sub-optimal) conditions. We recently published a study where we calculated the *Vibrionaceae* pangenome based on 124 genomes to study how the four pangene categories (core, softcore, shell, and cloud) are distributed on the genome (Sonnenberg *et al.* 2020). The analysis showed that core/softcore genes are typically found clustered around *ori1*, whereas shell and cloud genes densely populate terminus-proximate regions on Chr 1. On the chromid, genes are more randomly distributed, with no strong distribution pattern. On Chr 1, gene expression levels strongly correlate with distance to *ori1*, with higher expression levels around *ori1*. Interestingly, under slow-growing conditions all categories, except core genes, contribute to this pattern. This prompted us to question whether the observed gene distribution and expression patterns are specific to *Vibrionaceae*, or represent a general trend among bacteria with multipartite genomes.

The family *Pseudoalteromonadaceae* represents an excellent opportunity to study multipartite genomes, *e.g.*, how the genes are distributed and expressed, and its origin and evolution. As of March 2021, the Refseq database contains 53 complete *Pseudoalteromonadaceae* genomes. All genomes are bipartite and consist of one chromosome (3.1−4.9 Mb) and one chromid (0.6 − 1.8 Mb). Bosi *et al* (2017) calculated the *Pseudoalteromonas* pangenome based on 38 genomes (Bosi *et al.* 2017). Briefly, they described the pangenome as open and with a large percentage (80%) of unique genes. Furthermore, they estimated the last common ancestor (LCA) of *Pseudoalteromonas* to contain an estimated 2999 genes, compared to an average of 4245 genes in the present-day genomes, which supports that the genome has undergone a considerable expansion. More recently, Liao *et al* (2019) studied the evolution of the *Pseudoalteromonas* genome (Liao *et al.* 2019). Using a phylogenetic approach and timescale analysis, they showed that the chromosome and chromid have coexisted, probably since *Pseudoalteromonas* diverged from the putative LCA 500 million years ago. The chromid apparently originates from a megaplasmid that over time obtained essential genes (Médigue *et al.* 2005; Rong *et al.* 2016; Liao *et al.* 2019; Xie *et al.* 2021).

In summary, *Vibrionaceae* and *Pseudoalteromadaceae* represent the only two families from the gamma-proteobacteria class with multipartite genomes. Whereas the *Vibrionaceae* genome is well studied, the information on *Pseudoalteromadaceae* is scarce. In this study, we set out to gain insight into how pangene categories are distributed on *Pseudoalteromonadaceae* chromosomes and chromids, how genes are expressed relative to their genomic location, which genes can be regarded as hallmark genes of the chromid, and the origin and evolution of the chromid. We present data that support observations on gene distribution and global expression from other bacterial chromosomes, as well as data showing chromid-specific patterns that suggest specific roles of secondary replicons. Several pieces of evidence suggest the likely source of the chromid and its hallmark genes.

## Materials and methods

### Genome retrieval and gene annotation

A total of 25 *Pseudoalteromonas* genomes that were available at the onset of this project (mid 2019) at the National Center for Biotechnology Information (NCBI), were downloaded from the RefSeq database at NCBI (O’Leary *et al.* 2016) (see Supplementary File S1 for a complete list). The following genomes were excluded from the analysis: *Pseudoalteromonas atlantica* T6 (GCF_000014225.1) misplaced into *Pseudoalteromonas*, and later reclassified and renamed to *Paraglaciecola atlantica* T6. *P.* *atlantica* ECSMB14104 and *Pseudoalteromonas marina* ECSMB141043 are assembled into one contig, and the nature of their chromids could not be reliably resolved using Mauve. All genome sequences were re-annotated using RAST (Rapid Annotation using Subsystem Technology) version 2.0 (Aziz *et al.* 2008) with default settings. Mauve (Darling *et al.* 2004) was used to align genomes that were annotated with only one replicon.

### Phylogenetic analysis

Phylogenetic relationships between Alteromonadales genomes were inferred using the nucleotide sequences *gyrB, recA, rpoD*, *recN*, and *topA* as described earlier (Busch *et al.* 2019), and included the seven families *Alteromonadaceae, Colwelliaceae, Idiomarinaceae, Moritellaceae, Pseudoalteromonadaceae*, *Psychromonadaceae*, and *Shewanellaceae* (see Supplementary Figure S1 for complete phylogeny)*.* The nucleotide sequences were aligned using MUSCLE (Edgar 2004). Only unambiguously aligned positions were kept using BioEdit (Hall 1999), which resulted in a 9216 nt sequence alignment. MEGAX was used to generate a Maximum Likelihood (ML) tree, with the settings GTR (General Time Reversible) model, Gamma Distributed with Invariant (G + I), and Bootstrap with 1,000 pseudoreplicates (Kumar *et al.* 2018; Stecher *et al.* 2020). An ML-phylogeny of *Pseudoalteromonas* was based on 469 single-copy marker genes identified by EzTree (Wu 2018). The robustness of nodes was tested with a bootstrap analysis inferred from ML−GTR+G + I.

### Pangenome calculations

To classify the annotated *Pseudoalteromonas* protein sequences into four categories (core, softcore, shell, and cloud genes), we performed pangenome analysis using the software package GET_HOMOLOGUES (v3.1.0 (20180103) (Contreras-Moreira and Vinuesa 2013). The clustering algorithm MCL was used to cluster homologous protein sequences. The parameter “minimum percent sequence identity” was set to 50 and “minimum percent coverage in BLAST query/subj pairs” was set to 75 (default).

### Mapping of core, softcore, shell, and cloud genes on the *Pseudoalteromonas* genome

To study the distribution of core, softcore, shell, and cloud genes of *Pseudoalteromonas*, the chromosome and chromid sequences were divided into 4, 6, 8, 10, and 12 equally sized sections, with sector one starting at origin of replication (*gidA* on the chromosome and *parA* on the chromid). For each sector, the number of core, softcore, shell, and cloud genes were counted. The number of genes in each sector was then divided by the total gene number per replicon (probability of a gene belonging to a sector). The probability of a gene belonging to a sector given equal distribution between sectors was calculated for each of the 4, 6, 8, 10, and 12 sized sectors (1 divided on numbers of sectors). Then, the log10 ratio was calculated of the probability of a gene belonging to a sector divided by the probability given an equal distribution of genes between all sectors. Only a summary of the results when chromosomes and chromids are divided into six sectors are presented in the paper itself. The summary was made by calculating log10 ratio of: The probability of a gene belonging to a sector on average (Average #genes in a sector/Average total #genes)/The probability given an equal distribution of genes between all sectors (1/#sectors). See Supplementary File S2 for data. Kruskal–Wallis test and Dunńs test were used to perform pairwise comparisons of number of genes between all sections (see Supplementary File S3 for data).

### Gene expression analyses

RNA-seq datasets from *P*. *fuliginea* BSW20308 grown at three different temperatures, *i.e.*, 32° (BioSample accession no. SAMN06226833, SRR11593421, SRR11593421, and SRR11593422), 15° (sample no. SRR11593423, SRR11593424, and SRR11593425), and 4° (sample no. SRR11593426, SRR11593427, and SRR11593428) (Liao et al. 2019) were downloaded from the NCBI Sequence Read Archive (Leinonen *et al.* 2011) and analyzed. The quality of the reads was checked using FastQC (Andrews 2010). EDGE-pro v1.0.1 (Estimated Degree of Gene Expression in Prokaryotes) (Magoc et al. 2013) in Galaxy was used to align cDNA reads to the genome assembly (no. GCF_000310105.2) and estimate gene expression as reads per kilobase per million (RPKM) for all protein-coding sequences (CDS). The RPKM values were then used to calculate the log_2_ ratio RPKM CDS: RPKM median to make global expression maps for each of the three datasets. To identify which pangene categories contribute to the gene expression pattern, the chromosome was divided into “upper” and “lower” halves, and the chromid was divided into “upper” and “lower” halves, as well as “right” and “left” halves, and the RPKM median value for each pangene category was calculated (Supplementary File S4).

### BLASTp searches

Homologs of chromid hallmark genes were identified by BLASTp when using the nonredundant database, and excluding the *Pseudoalteromonadaceae* family (taxid: 267888), with the thresholds: *e*-value < 1e^−15^, sequence identity >30% and sequence coverage >70% (see Supplementary File S6).

### Statistical analysis

Statistical analysis was performed using R in RStudio (RStudio Team 2021). Kruskal–Wallis test and Dunn’ s test were used to perform pairwise comparisons of number of genes in replicon sections. The tests were chosen because the data did not follow a normal distribution, and sample sizes were low. R’s Kruskal.test() function for the rank-based nonparameteric Kruskal–Wallis test and the dunn.test() function for *post hoc* Dunn’s test was used (see Supplementary Files S2 and S3 for data). Significant difference of gene expression between replicon halves and replicons was performed using R’s wilcox.test() function for unpaired Wilcoxon signed-rank tests (see Supplementary File S4 for data). For all analyses, *P*-values were Bonferroni corrected for multiple comparisons using R’s p.adjust() function.

## Results

### *Pseudoalteromonadaceae* branches off from families with monopartite genomes

**Figure 1:**
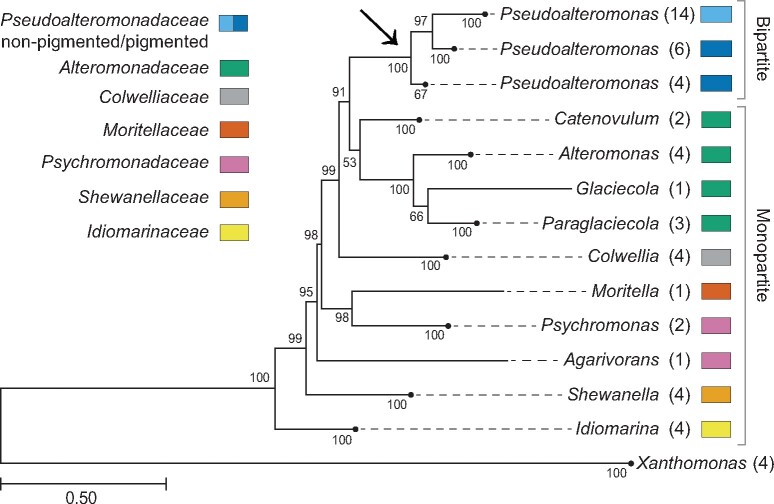
Summary of an ML-phylogenetic tree showing evolutionary relationships between Alteromonadales families. See Supplementary Figure S1 for complete tree. Multipartite genomes are restricted to *Pseudoalteromonadaceae*, which is placed at the crown of the tree and branches off from families containing monopartite genomes. The arrow highlights the LCA of *Pseudoalteromonadaceae*, and the likely point of origin of the *Pseudoalteromonadaceae* chromid. The color scheme shows family affiliation of genera. Numbers of strains included in collapsed nodes are shown in parentheses. Bootstrap values at the nodes were calculated using the ML method, and the GTR+G + I model, with 1000 replicates.

Figure 1 shows the overall phylogenetic relationships between bacterial families and genera that form the order Alteromonadales (see Supplementary Figure S1 for complete phylogeny). The ML-tree (GTR+G + I model) was based on the concatenated nucleotide sequences of *gyrB, recA, rpoD*, *recN*, and *topA* from selected bacteria from the seven families *Alteromonadaceae, Colwelliaceae, Idiomarinaceae, Moritellaceae, Pseudoalteromonadaceae*, *Psychromonadaceae*, and *Shewanellaceae.* The analysis shows that each genera and family forms well-supported monophyletic groups, similar to previous studies (Williams et al. 2010; Martin *et al.* 2015). Notably, the family *Pseudoalteromonadaceae* (includes only the genus *Pseudoalteromonas*), which exclusively contains bacteria with multipartite genomes, branches off from the monopartite genome-containing clades as a crown group together with its sister *Alteromonadaceae*. None of the bacteria outside of *Pseudoalteromonadaceae* contain multipartite genomes. These two observations strongly support that the chromid was acquired by the most recent LCA of *Pseudoalteromonadaceae*, likely in a single event (indicated with an arrow in Figure 1). A single origin of the chromid is supported by a phylogenetic analysis that showed congruent phylogenies between the chromosome and chromid (Liao *et al.* 2019). Finally, two separate estimates of time since divergence suggest that *Pseudoalteromonadaceae* branched off approximately 500, and 502–378 million years ago (Liao *et al.* 2019; Xie et al. 2021). Compared to *Vibrionaceae*, which also exclusively contains multipartite genomes, the birth of *Pseudoalteromonadaceae* is relatively recent. The time since divergence of *Vibrionaceae* is estimated to approximately 1100–900 million years ago (Xie et al. 2021).

### The *Pseudoalteromonadaceae* pangenome contains 1399 core genes

The definite point of origin of the *Pseudoalteromonadaceae* chromid prompted us to study the multipartite genome in more detail, *e.g.*, to identify where the chromid replicon was acquired from, and how it has evolved after its acquisition. We used a pangenome approach as previously described (Sonnenberg *et al.* 2020). Briefly, available complete genomes were downloaded and re-annotated using RAST (Aziz *et al.* 2008). Genome datasets were then used to cluster orthologous groups of protein sequences based on the MCL algorithm (Van Dongen 2000) in GET_HOMOLOGUES (Contreras-Moreira and Vinuesa 2013). By using 25 available complete *Pseudoalteromonadaceae* genomes, which were available by the onset of the calculations (see Supplementary File S1 for complete list), we found a total of 24,991 clusters. The clusters were sub-categorized into 1399 core (encoded by all 25 genomes), 1606 softcore (encoded by ≥23 genomes), 7688 shell (encoded by ≤22 and ≥3 genomes), and finally 15,697 cloud (encoded by ≤2 genomes). This result is comparable to the calculations reported by Bosi *et al* (2017), based on 38 *Pseudoalteromonas* genomes (mostly draft genomes), which identified a total of 22,530 clusters, sub-divided into 1571 core (encoded by all 38 genomes), 2901 shell (encoded ≤37 and ≥2 genomes), and 18,058 cloud (encoded by one strain) (Bosi *et al.* 2017).

### The distribution of core/softcore genes on the *Pseudoalteromonas* chromid strongly correlates with the direction of replication

To establish the distribution pattern of *Pseudoalteromonas* pangenes, we mapped all representative genes from the four pangene categories core, softcore, shell, and cloud to their chromosomal or chromidal locations. First, chromosome and chromid sequences were divided into 4, 6, 8, 10, or 12 equally sized sectors (or bins), with sector one starting from the origin of replication and proceeding clockwise. For each sector, the number of genes from each category were counted. At least for primary replicons, previous data from other bacterial families (Comandatore *et al.* 2019; Kopejtka *et al.* 2019; Sonnenberg *et al.* 2020), have shown that core/softcore genes densely populate regions that are replicated early in the replication cycle, and we hypothesized that *Pseudoalteromonas* would generate a similar distribution pattern. Notably, a recent study showed that most *Pseudoalteromonas* chromids are replicated unidirectionally, except for *Pseudoalteromonas spongiae* and *Pseudoalteromonas piratica* chromids, which are replicated bidirectional (Xie et al. 2021).

**Figure 2:**
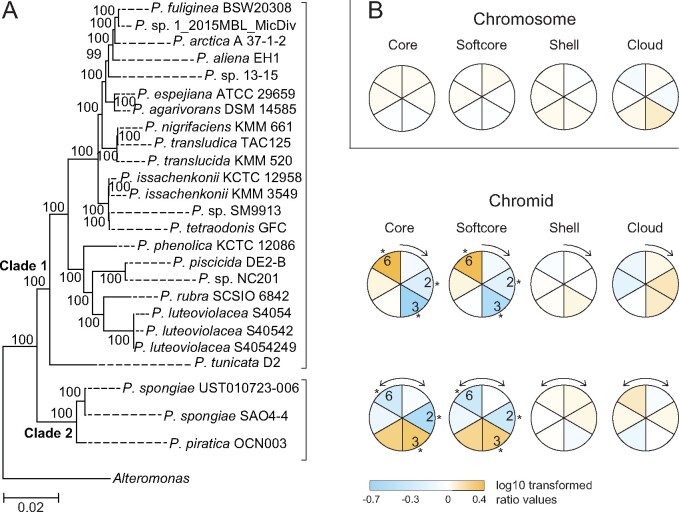
Heatmaps of distribution of core, softcore, shell, and cloud genes in *Pseudoalteromonas* genomes. Genes were placed into one of six equally sized sectors of chromids (A) or chromosomes (B), with sector 1 starting at the origin of replication (12 o’clock). Unidirectionally replicated chromids are found in species that belong to Clade 1, as shown in the ML-phylogeny (GTR+G + I model, 1000 replicates), whereas bidirectionally replicated chromids belong to representatives of Clade 2. Heatmaps are based on log _10_ ratio values of the probability of a gene belonging to a sector on average divided by the probability given an equal distribution of genes among all sectors. Positive values (shades of orange) suggest that gene categories are overrepresented, whereas negative values (shades of blue) suggest underrepresentation. Asterisks indicate significant over- or under-representation of gene categories using Dunńs test *P*-value ≤ 0.05 (see Supplementary Files S2 and S3 for more details). The phylogenomic tree is based on 469 single marker genes identified by EzTree (Wu 2018). Bootstrap values at the internal nodes were inferred from a ML−G + I analysis.

Figure 2A shows the result mapped onto a *Pseudoalteromonas* ML-phylogeny based on 469 single-copy marker genes identified by ezTree (Wu 2018). Heatmaps summarize the result for Clade 1 (unidirectional replication of chromid), and for Clade 2 (bidirectional replication of chromid). The heatmaps are based on average values from the 25 analyzed genomes (see Materials and Methods). Only data for chromids divided into 6 sectors are shown (see Supplementary File S2 for all datasets). The Kruskal–Wallis and the Dunńs *post hoc* tests were used to identifying significant over- or under-representation of gene numbers between all pairs of sectors (see Supplementary File S3). The main finding is that core/softcore genes densely populates late replicating chromidal sectors, regardless of if chromids are replicated uni- or bi-directionally, which is surprising and opposite of what we expected. Specifically, for unidirectionally replicated chromids (Clade 1), core/softcore genes are strongly overrepresented in sector 6, and underrepresented in sectors 2 and 3. For bidirectionally replicated chromids (Clade 2), core/softcore genes are strongly overrepresented in sectors 3 and 4, and underrepresented in sectors 2 and 6.

None of the pangene categories are, in contrast, significantly over- or under-represented in specific regions of the chromosome. Instead, core/softcore genes are only weakly overrepresented in sectors 1, 5, and 6 (near origin of replication), shell genes are weakly overrepresented in sectors 3 and 4 (near terminus of replication), and cloud genes are weakly overrepresented in sector 3. The general pattern is therefore similar to, but less pronounced than what has been reported for *e.g.*, *Vibrionaceae* (Sonnenberg *et al.* 2020), *Klebsiella pneumonia* (Comandatore *et al.* 2019), and *Rhodobacteraceae* (Kopejtka *et al.* 2019).

In summary, by dividing the two *Pseudoalteromonas* replicons into 4−12 sectors and calculating the log_10_ ratio of the probability of a gene belonging to a sector divided by the probability given an equal distribution, we showed that core/softcore genes are significantly overrepresented in late replicating sectors of the chromid, regardless of how the chromid is replicated, *i.e.*, unidirectionally (Clade 1 strains) or bidirectionally (Clade 2 strains). Chromosomal genes are in contrast more evenly distributed into each sector of the replicon.

### Gene dosage is in effect on the *Pseudoalteromonas fuliginea* BSW20308 chromosome, but not on the chromid

It is well established that the copy number of *ori*-proximate genes can increase during rapid growth due to the formation of multiple concurrent replication forks, which in turn result in multiple copies of the replicon (*e.g.*, a chromosome), and increased gene expression. This is known as the “gene dosage effect.” To date, this has been described for the *Vibrionaceae* Chr 1 (Rasmussen *et al.* 2007; Srivastava and Chattoraj 2007; Dryselius *et al.* 2008; Toffano-Nioche *et al.* 2012), *Escherichia coli*, *Bacillus* *subtilis*, and *Streptomyces* (Couturier and Rocha 2006; Lato and Golding 2020). To establish if a gene dosage effect is in play in *Pseudoalteromonas* (for the chromosome, the chromid or both), we downloaded data from one of two available RNA-seq experiments stored at the NCBI Sequence Read Archive (Leinonen *et al.* 2011). In the selected experiment, *P*. *fuliginea* BSW20308 was grown in Difco marine broth 2216 and harvested at 4° (lowest temperature with growth), 15° (optimal growth), or 32° (maximum temperatures with growth) (Liao *et al.* 2019). These datasets, therefore, provide an excellent chance to test gene dosage effects at fast and slow growth, which is highly relevant because gene dosage has been reported to be particularly strong at rapid growth (and therefore rapid replication). The three RNA-seq datasets (each in triplicates) were analyzed as previously described (Sonnenberg *et al.* 2020). Briefly, cDNA reads were mapped onto the *P. fuliginea* BSW20308 genome (assembly no. GCF_000310105.2) and reads RPKM was calculated for all protein CDS.

**Figure 3:**
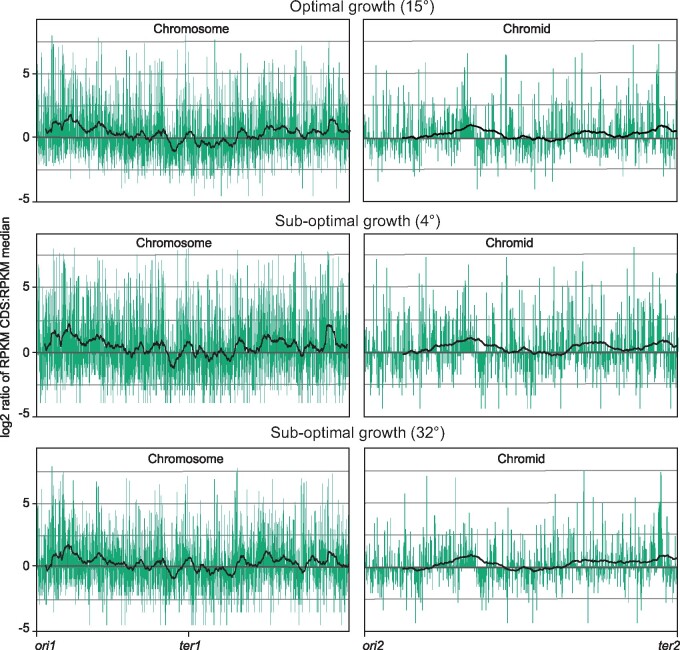
Global expression maps of *P. fuliginea* BSW20306 chromosomal and chromid genes centered on the median. Data points (log_2_ ratio RPKM CDS: RPKM median) for each CDS are shown, as well as a trend line averaged over a sliding window of 100 data points. The temperatures 4° and 32° corresponds to sub-optimal growth conditions and 15° corresponds to optimal growth conditions.

Figure 3 shows global expression maps of the chromosome and chromid when *P*. *fuliginea* BSW20308 was grown at 4°, 15°, or 32°. Data points (log_2_ ratio RPKM CDS: RPKM median) are centered around the RPKM median. Moreover, for each plot a trend line averaged over a sliding window of 100 data points was added to show the overall direction of the data. Expression from the primary replicon (*i.e.*, the chromosome) is trending downwards starting from *ori1* and ending at *ter1*, with a low point at position 1,734,472. In other words, RPKM values are typically higher on the upper half compared to the lower half, which is expected if gene dosage is in effect on a bidirectionally replicated chromosome. This finding is strongly supported by the Wilcoxon signed-rank test (*P*-adj ≤ 0.05) (see Supplementary File S4). Expression from the chromid is elevated in the intermediate and late replicating regions. This expression pattern is opposite of what is expected if gene dosage is in effect on a unidirectionally replicated chromid, as in this case. If gene dosage was in effect then overlapping replication cycles would increase the number of DNA copies on the chromidal half (*i.e.*, the “right” half) which is replicated first. The Wilcoxon test does not, however, support significant differences in gene expression neither between upper and lower halves, or left and right halves (see Supplementary File S4).

In summary, we found that gene dosage appears to be in effect on the *Pseudoalteromonas* chromosome, but not on the chromid. This applies for all three tested temperatures, 4°, 15°, or 32°, which can be regarded as the minimum, optimum or maximum growth temperatures, respectively.

### All pangene categories contribute to higher gene expression on the upper half of the *Pseudoalteromonas* chromosome under optimal growth temperature

To establish which pangene categories contribute to the gene dosage effect on the *P. fuliginea* BSW20308 chromosome, we calculated the RPKM median value for each pangene category (Table 1). The Wilcoxon signed-rank test strongly support (*P*-adj ≤ 0.05) that all four pangene categories contribute, when *P*. *fuliginea* BSW20308 is cultured at optimal conditions (15°). Interestingly, when grown at sub-optimal conditions (4° and 32°), the same test identifies only shell genes as significant contributors (see Table 1). The RNA-seq data further shows that RPKM median values of core and softcore genes are generally higher than that of shell and cloud genes (see Supplementary File S4), and this is valid for all three datasets except for the chromosome when grown under 32°. As expected, RPKM values are generally highest when grown at optimal temperature (15°), slightly lower at 4° and lowest at 32°. Overall, expression from chromosomal genes is higher compared to chromidal genes at 15° (RPKM median = 45 and 31, *P*-adj = 0.0), 4° (RPKM median = 30 and 20, *P*-adj = 0.0), and 32° (RPKM median = 22 and 20.5, *P*-adj = 0.043).

**Table 1:** Comparison of gene expression levels for pangenes located on the upper or lower halves of the *P*. *fuliginea* BSW20308 chromosome

	**Optimal growth conditions (15**°**)**				**Sub-optimal growth conditions (4**°**)**				**Sub-optimal growth conditions (32**°**)**			
	Core	Softcore	Shell	Cloud	Core	Softcore	Shell	Cloud	Core	Softcore	Shell	Cloud
Upper half*a*												
Q_1_	41	39	26	20	30	29	11	7	20	19	10	8
Q_2_	84	82	43	37	77	74	24	15	41	39	18	15
Q_3_	253	245	94	70	290	267	67	30	115	107	43	34
Max	11,063	11,063	134,285	37,786	48,320	48,320	169,723	53,846	5,208	5,208	35,549	8,083
Lower half*a*												
Q_1_	29	28	16	13	24	21	7	5	18	17	8	6
Q_2_	65	64	28	26	66	65	16	12	40	38	14	13
Q_3_	185	181	59	55	267	232	46	32	97	94	32	27
Max	11,172	11,172	19,840	1,635	6,927	6,927	17,176	578	2,047	2,047	4,884	523
*P*-value Q_2_*b*	0.00	0.00	0.00	0.00	0.08	0.07	0.00	0.15	0.40	0.40	0.00	0.20

a Q_1_ is the RPKM value at the first quartile. Q_1_ is defined as the middle number between the smallest number and the median (*i.e.*, the second quartile Q_2_), if the data numbers (in this case RPKM values) are ordered from smallest to largest. The third quartile (Q_3_) is the middle value between the median (Q_2_) and the maximum (Max) value.

b Adjusted *P*-values from Wilcoxon signed-rank test, to test if Q_2_ values (median) of genes located on the upper half of the chromosome are significantly different from Q_2_ values of genes located on the lower half. Values below 0.05 are considered significant.

To summarize, under optimal growth conditions all four pangene categories contribute to higher gene expression on the upper part of the chromosome, whereas only shell genes contribute under sub-optimal conditions. As expected, absolute RPKM values are generally highest for core and softcore genes, and the median RPKM value for the chromosome is significantly higher than that of the chromid.

### The *Pseudoalteromonas* chromid originates from an ancestral plasmid similar to those found in extant species of Alteromonadales

To investigate where the *Pseudoalteromonas* chromid originates from, we performed BLASTp searches using the chromid ParA, ParB, and RepA proteins as queries against the nr. database. The tripartite ParA-ParB-*parS* system consists of a ParA ATPase, a ParB CTPase and DNA-binding protein, and a centromere-like *parS* site, and is responsible for faithful segregation of replicons during cell growth and division in approximately three quarters of bacteria (Jalal *et al.* 2020). RepA is the replication initiator protein in *Pseudoalteromonas* chromids (Xie et al. 2021). The fundamental function of the partitioning system and the replication initiator protein, together with their widespread distribution in Bacteria and Archaea, make ParA, ParB, and RepA excellent candidates for finding clues to the origin of the chromid.

ParA and ParB BLASTp searches both identified homologs from draft genomes of *Rheinheimera* and *Catenovulum* as best hits (*e*-values = 0/0, Identities = 39%/41%; see top 20 list in Supplementary File S5). We believe that these hits represent auxiliary chromosomal ParA and ParB sequences originating from integrated plasmids. Following the top hits are a number of high-scoring matches against plasmids from *Pseudoalteromonas*, *Shewanella*, *Vibrio*, *Catenovulum*, *Alteromonas*, and *Glaciecola*. RepA BLASTp identified *Rheinheimera* and *Shewanella* draft genomes and *Catenovolum sediminis* plasmid as best hits, followed by *Aeromonas* plasmids. The BLASTp results therefore strongly suggest that the *Pseudoalteromonas* chromid originates from an ancestral plasmid, or possibly a megaplasmid, similar to those found in extant Alteromonadales species. Moreover, the relatively large size of today’s *Pseudoalteromonas* chromids suggests that the acquired plasmid or megaplasmid has accumulated a vast number of genes that over time evolved into an in-dispensable replicon. A similar origin has been suggested for the *Vibrionaceae* chromid (*i.e.*, Chr 2) (Heidelberg *et al.* 2000; Fournes *et al.* 2018) and other chromids (Harrison *et al.* 2010; diCenzo *et al.* 2019).

### More than half of the chromid hallmark genes in *Pseudoalteromonas* originates from the ancestral gene pool of Alteromonadales

Given that the *Pseudoalteromonas* chromid originates from an ancestral plasmid, then new questions emerge. For example, which type of genes are associated with chromids? Potential genetic sources could be genes from the *Pseudoalteromonas* chromosome, and/or genes from chromosome, chromid or plasmid DNA from closely or distantly related bacteria. To address this, we used the results from our pangenome analysis of *Pseudoalteromonas* genomes, and calculated the number of genes from each pangene category that are associated with the chromid. Any gene that was found at least once on a chromid was included. We found 164 core, 746 softcore, 2097 shell, and 4790 cloud genes, in total 7633 genes.

To find the genetic source of chromid genes we carefully selected a set of proteins and used them as queries in BLASTp searches. Of the 164 core genes only 78 are always located on the chromid (see Supplementary File S6 for complete list of genes). These are hereafter referred to as “chromid hallmark genes.” Their ubiquitous presence on chromids support that they were acquired by the LCA, before diversification of *Pseudoalteromonas* took place (see arrow in Figure 1). Interestingly, about half (31) of the chromid hallmark genes are found clustered close to the replication terminus, and include genes and operons involved in histidine biosynthesis (*hisIEFAHBCDG*), DNA binding protein (*hupB*), acetolactate synthase (*ilvBH*), biopolymer transport system (*tonB-exbB-exbD)*, and cell division (*minCDE*) (see Supplementary File S6 for more information). For the ancestral plasmid to be maintained and become part of the stable genome we see today, the chromid hallmark genes probably provided a selective advantage. We, therefore, regard these genes as great candidates for studying the origin of early chromid genes. All chromid hallmark proteins were used as queries in BLASTp searches (Supplementary File S6). In total, 42 (58%) of the proteins produced the highest scoring matches to sequences from Alteromonadales (after excluding matches from *Pseudoalteromonas*), followed by Chromatiales (11%), Vibrionales (10%), and Oceanospirillales (8%). This suggests that more than half of the current hallmark genes originates from the ancestral gene pool of Alteromonadales, whereas the remaining genes were acquired from diverse sources, mostly other gamma-proteobacteria.

### The *Pseudoalteromonas* chromid contains a large number of genes with roles in iron uptake and homeostasis

A surprisingly large number of genes associated with iron-acquisition and homeostasis are located on the *Pseudoalteromonas* chromid. For example, in all 25 genomes, two *bfr* genes that encode bacterioferritin are located on the chromid, often flanked by *bdf* (encodes bacterioferritin-associated ferredoxin) and *iutA* (aerobactin siderophore receptor gene)*.* Moreover, two complete *tonB-exbB-exbD* systems are found in all genomes, one on the chromid and one on the chromosome. And, in addition to *iutA*, six other TonB-dependent siderophore receptor genes are associated with the chromid, including *fhuA* (ferrichrome), *fhuE* (coprogen, rhodoturulate), *viuA* (vibriobactin), *fepA* (enterobactin), *desA* (deferoxamine B), and *vctA* (enterobactin). As previously reported for *Pseudoalteromonas tunicata* D2 (Thomas *et al.* 2008), a complete siderophore biosynthesis gene cluster is yet to be found in any of the *Pseudoalteromonas* genomes, even though they carry a relatively large number of siderophore receptor genes. This suggests that *Pseudoalteromonas* are “cheaters” *i.e.*, they have siderophore receptors on their surface with affinity to compounds produced by other bacteria (Payne *et al.* 2016). This mechanism is used as a strategy to avoid being discriminated against by other bacteria in the constant struggle between microorganisms to acquire iron.

## Discussion

The number of studies on multipartite bacterial genomes has steadily increased along with the number of available finished genomes in public databases. As of March 16th 2021, there are 306,881 bacterial genome assemblies listed in the NCBI genome database, of which 22,910 are denoted as “complete” (7.5%). However, whereas some phyla are well represented, with *e.g.*, 57% of complete genomes belonging to Proteobacteria, and 34% belonging to Terrabacteria, most groups of bacteria are poorly represented, or not represented at all. Opportunities for doing studies on many gap-free multipartite genomes from single families are therefore rare. *Pseudoalteromonas* represents one of these rare cases. The chromid appears to originate from a relatively recent event that can be placed at a specific branch on the evolutionary tree with high confidence. We have therefore taken the opportunity to study the *Pseudoalteromonas* genome, and mapped how different gene categories are distributed and expressed in order to shed light on possible mechanisms that have shaped the chromosome and chromid.

We found that the *Pseudoalteromonas* genome partly confirms observations from other families, *e.g.*, that core/softcore genes appear more frequent around *ori1*, and shell (accessory) genes occur more frequent around *ter1* (Figure 2)*.* We recently reported a similar strong correlation for *Vibrionaceae* Chr 1 (Sonnenberg *et al.* 2020). Using a slightly different pangenomic approach Comandatore et al. (2019) found a similar distribution pattern in *K.* *pneumonia*, whereas Kopejtka et al. (2019) reported a more complex picture with 22 species from *Rhodobacteraceae* showing clustering of core genes close to *oriC*, and eight species showing clustering around *ter* (Comandatore *et al.* 2019; Kopejtka *et al.* 2019). One plausible hypothesis is that core/softcore genes, which are associated with essential cell processes, are typically overrepresented around *oriC* because their gene products are of high demand during fast growth (Slager and Veening 2016). The rationale is that several concurrent initiations of replication from *oriC* results, on average, in higher “doses” of *oriC*-proximate genes. In turn, this leads to increased gene expression (the “gene dosage” effect) (Couturier and Rocha 2006). Analyses of *V. natriegens* and *A. salmonicida* (Sonnenberg *et al.* 2020), *Salmonella enterica* (Garmendia *et al.* 2018), and eleven bacterial data sets of diverse origin (Lato and Golding 2020) all confirmed that overall expression decreases with increasing distance to *oriC*. Our current analysis of a *P. fuliginea* BSW20308 RNA-seq data replicates a similar pattern (Figure 3).

The distribution pattern for chromid genes is in contrast very different, and perhaps more difficult to explain. For hitherto unknown reasons, the presence of core genes strongly correlates with distance to *ter2*. Interestingly, a recent study concluded that chromids belonging to the *P. spongiae* group are replicated bidirectionally, whereas chromids in all other *Pseudoalteromonas* are replicated unidirectionally (Xie et al. 2021). Accordingly, in bidirectionally replicated chromids *ter2* is located at 6 o’clock, and in unidirectionally replicated chromids *ter2* is located at 12 o’clock. The fact that core genes are overrepresented at *ter2* suggests that the genes are typically found in chromid sections that are replicated in the final part of the replication cycle, a situation that is opposite to that of *e.g.*, the *Pseudoalteromonas* and *Vibrionaceae* chromosomes where the gene dosage effect is in play. Gene dosage is apparently not in effect in *Pseudoalteromonas* chromids which suggests that we need another explanation for the clustering of core genes.

We can only speculate on why core/softcore genes tend to be located at *ter2*, but an intriguing possibility that we recently introduced for *Vibrionaceae* (Sonnenberg *et al.* 2020), is that the genomic distribution of gene categories is directly linked with how genes are organized into subcellular locations. In *V. cholerae*, Chrs 1 and 2 are longitudinally organized, with *ori1* located at the old pole, *ter1* and *ter2* located at the new pole, and *ori2* placed at the cell center (Fogel and Waldor 2005; Srivastava and Chattoraj 2007; David *et al.* 2014). Together, the data from *V. cholera* suggests to us that core/softcore and shell/cloud genes are enriched at two separate cellular locations, *i.e.*, at the old and new poles, respectively. Given that this hypothesis is correct, then it is plausible that a similar pattern/mechanism is in play in *Pseudoalteromonas*. It should be stressed that this remains per today a hypothesis, although the cytoplasmic position of individual gene loci have previously been successfully predicted based on the spatially organization of chromosomes (reviewed in Surovtsev and Jacobs-Wagner 2018). Moreover, for the hypothesis to be valid for *Pseudoalteromonas* there is an additional prerequisite that must be fulfilled: *ter2* is located at 6 or 12 o’clock (relative to *ori2*) depending on the replication mechanism that is in play (uni- or bi-directional). If *ter2* is deciding the subcellular destination of *ter2*-proximate core genes then they should, in principle, be located at the same subcellular compartment regardless of the replication mechanism. If, however, *ori2* is the decisive genetic component for intracellular positioning of the chromid, then *ter2/*associated core genes will be located at different spatial places depending on the replication mechanism (and positioning of *ter2* relative to *ori2*). Unfortunately, there is currently no evidence to suggest how *Pseudoalteromonas* cells are spatially organized intracellularly with regards to their chromatin. We note that the Min system (*minCDE*), which represents one of the best-studied proteins involved in cellular self-organization (reviewed by Wettmann and Kruse 2018), is located in the vicinity of *ter2*, but the significance of this is currently unclear to us.

Our results suggest that today's chromid in *Pseudoalteromonas* originated from a plasmid that was acquired in a single event in the LCA of this family. By comparing the chromid ParAB with database sequences we found that the best hits belong to plasmids found within today’s representatives of Alteromonadales (Supplementary File S5). An early acquisition of chromid is further supported by congruent phylogenies of the chromosome and chromid, which support that the two replicons have coexisted since the LCA of *Pseudoalteromonas* (Liao *et al.* 2019; Xie et al. 2021). Given an early acquisition of a plasmid or megaplasmid, what then were the main driving forces for retaining and expanding the replicon size into a relatively large chromid? diCenzo *et al.* (2019) recently proposed that the main advantage with secondary replicons, is that they enable increased genetic flexibility and potential to acquire new genetic material (diCenzo *et al.* 2019). As a result, the bacterium is better suited to take advantage of new niche opportunities. It is an appealing concept, and several pieces of evidence from our study support the hypothesis. Perhaps the most compelling evidence comes from our pangenome calculations that identify the *Pseudoalteromonas* chromid as open and extremely flexible. A total of 7633 genes are associated with the chromid, which is approximately 10x greater that the number of genes encoded by individual chromids (553–1567 genes; median = 781). Moreover, chromid genes are generally expressed at a lower level, which have been suggested to increase the likelihood of newly acquired genes to be retained in the genome (Park and Zhang 2012; diCenzo *et al.* 2019). This is likely because highly expressed and mostly more critical genes on the chromosome are not disrupted, which then leads to less fitness cost for the bacterium. As a final piece of the puzzle, the vast majority of chromid genes in *Pseudoalteromonas* belong to the categories shell or cloud (see Supplementary File S2), which provides further support for the hypothesis that new genes are preferentially maintained on the chromid and thus increases the genetic plasticity of the *Pseudoalteromonas* genome.

To summarize, we provide data showing that *Pseudoalteromonas* core/softcore genes are weakly overrepresented at *oriC*-proximate regions, whereas shell/unique genes are weakly overrepresented around *ter1*. This distribution fits with patterns reported earlier for other bacteria (Comandatore *et al.* 2019; Kopejtka *et al.* 2019; Sonnenberg *et al.* 2020). Similarly, we found that gene expression is trending downwards with increasing distance to *oriC*, which also fits a general pattern among many bacteria (Garmendia *et al.* 2018; Lato and Golding 2020; Sonnenberg *et al.* 2020). For secondary replicons, the situation appears more complex. Here, the distribution pattern for pangene categories, as well as global expression maps, vary greatly among the studied bacteria. Perhaps the reason for the apparent lack of general trends is a direct result of the specialized roles of chromids, which have been shaped by the acquired and retained set of (mostly shell/unique) genes. Finally, we hypothesize that the gene distribution patterns reported by us and others are directly linked to how the DNAs are organized intracellularly, such that different pangene categories are enriched at separate subcellular locations based on their specialized biological functions.

## Data availability

Supplemental data are available in Supplementary Figure S1 and Supplementary Files S1–S6. Supplementary Figure S1 shows phylogenetic relationships between Alteromonadales families. A list of all 25 *Pseudoalteromonas* genomes used in this study are available in Supplementary File S1. Supplementary File S2 contains distribution data of core, softcore, shell, and cloud on *Pseudoalteromonas* chromosome and chromid divided into 4, 6, 8, 10, and 12 sectors. Supplementary File S3 contains statistical analysis of pairwise comparisons of number of genes between sectors (Kruskal–Wallis and Dunńs test). Statistical analysis of gene expression of *P. fuliginea* BSW20306 chromosome and chromid using Wilcoxon signed-rank test is available in Supplementary File S4. Supplementary File S5 contains BLASTp results with chromid ParA, ParB, and RepA as queries. A list of chromid hallmark genes and BLASTp results is available in Supplementary File S6. Supplemental material is available at figshare: https://doi.org/10.25387/g3.14900463.

## Funding

This work was supported by the UiT The Arctic University of Norway. The publication charges for this article have been funded by a grant from the publication fund of UiT The Arctic University of Norway. The funder had no role in study design, data collection, and interpretation, or the decision to submit the work for publication.

## Conflicts of interest

The authors declare that there is no conflict of interest.
